# Follicular Dendritic Cell Tumor in Liver: A Case Report and Collective Review

**DOI:** 10.4021/gr2010.05.206w

**Published:** 2010-05-20

**Authors:** Chee-Hwee Lee, Yu-Shan Yen, Yan-Shen Shan, Pin-Wen Lin

**Affiliations:** a Division of General Surgery, Department of Surgery, National Cheng Kung University Hospital, College of Medicine, National Cheng Kung University, Taiwan; b Department of Pathology, National Cheng Kung University Hospital, College of Medicine, National Cheng Kung University, Taiwan

**Keywords:** Follicular dendritic cells, Hepatic tumor

## Abstract

Follicular dendritic cells (FDC) are a subset of the immune system and present in the germinal centers of lymphoid follicles in the spleen and lymph nodes. They are functionally as antigen-presenting cells and thus improving the quality of the humoral immune response. Follicular dendritic cell tumor is rare but considered low-grade sarcoma. Including our case, there were totally 17 cases with FDC tumor involved the liver from Medline. In these cases, the mean age was 47 years old, ranged from 19 to 82. It was much more common in females than in males (13:4). The clinical manifestations of these patients included abdominal discomfort, palpable mass, weight loss and malaise. The average size of the tumor was 11 cm. Most of the FDC tumors were associated with Epstein-Barr virus expression, 13/17 (76.5%). Surgical resection remains the mainstay of the treatment.

## Case Report

**Figure 1:**
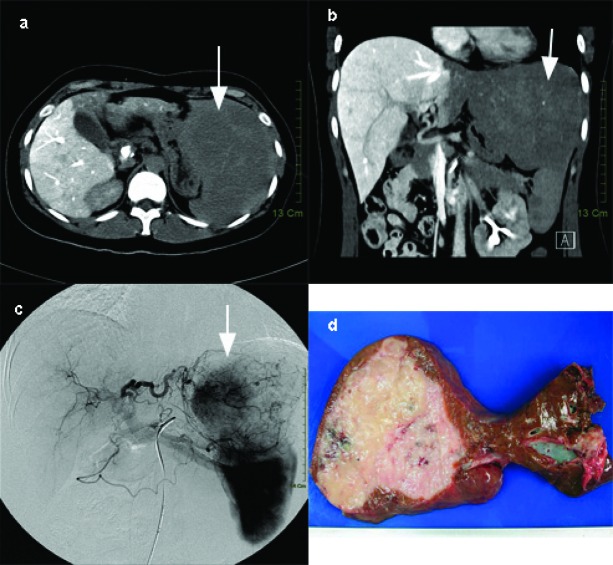
(a) Enhanced abdominalCT of horizontal view which revealed huge hepatic hypervascular mass of about 14 cm in size (arrow). (b) Enhanced abdominalCT of coronal view which revealed huge hepatic hypervascular mass of about 14 cm in size.(arrow). (c) Angiography revealed the mass was mainly supplied by the left hepatic(arrow). (d) Macroscopic view of the resected follicular dendritic cell tumor of liver as a round softmass and on cut section shows whitish to pinkish surface without centralnecrosis.

**Figure 2:**
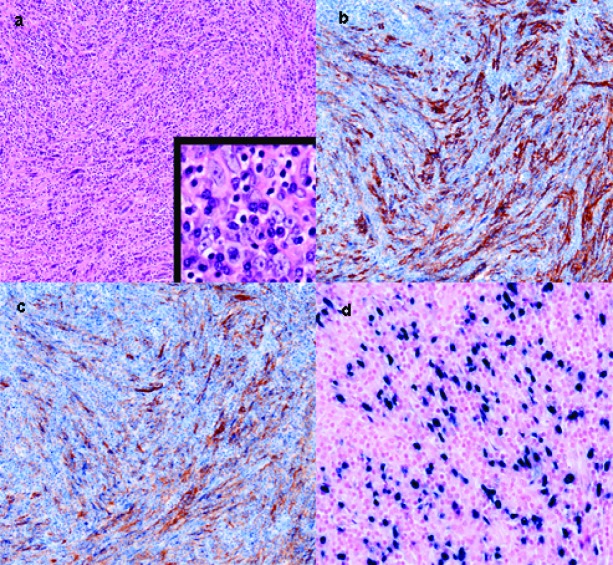
(a) Microscopic appearance of follicular dendritic cell tumor. At low magnification, the tumorcells in whorls pattern show hypechromatism and pleomorphism, and interspersedplasma cells and lymphocytes. Higher magnification demonstrates slight eosinophilic cytoplasm, rare mitosis, and indistinct cell borders with granular chromatin and small nucleoli. (b) Immunohistochemical staining for CD 21, the spindle-shaped tumor cells show long cytoplasmic processes which stain brownish. (c) Immunohistochemical staining for CD 35, a marker for follicular dendritic cells reveals tumor cells stained brownish. (d) Positive expression of EBV nuclear RNA on tumor cells by in situ hybridization.

A 36 year-old female complained of persisted dull pain over epigastric area for 2 months. No associated symptoms such as heartburn, nausea, and vomiting can be elicited. She visited local clinic and a mass was palpated in the left upper quadrant area. At the same time, abdominal sonography showed a huge hepatic mass at left lobe of liver. So, she received examination of abdominal computed tomography (CT) thereafter. Abdominal CT (Fig. 1a, b) revealed a huge left hepatic mass with a size of 20 x 15 cm. She was then transferred to our institution for further management. Initial laboratory data were only notable for a decreased hemoglobin level of 9.7 g/dL and a mildly elevated level of alkaline phosphatase (139 U/L). The serum marker of hepatitis B or hepatitis C was also negative. Tumor markers, including serum carcinoembryonic antigen (CEA), CA19-9, and α-fetoprotein level (AFP) were within normal value. Angiography revealed this huge hepatic mass was supplied by left hepatic arterial (Fig. 1c). After studies, she received operation for excision of the tumor. The excised liver measured 20 x 15 x 10 cm and weighed 793 gram. The cutting surface showed a huge soft mass of size 14 x 12 x 6 cm with whitish to pinkish surface (Fig. 1d). There was no central necrosis in the tumor. The tumor margin was greater than 5 cm away from the nearest section margin. There was no cirrhotic change at the remaining part of liver parenchyma. Microscopically, the lesion was mainly composed of hyperchromatic and mild pleomorphic spindle cells admixed with many reactive plasma cells and lymphocytes. These neoplastic cells formed fascicles, storiform and whorls pattern. They had plump cytoplasm and indistinct cell borders with vesicular or granular chromatin and small nucleoli. Some giant cells and fibrous bands were seen but rare mitotic figures were found within the tumor (Fig. 2a). These tumor cells were positive for expression of CD21 and CD35 by immunohistochemical staining and positive for expression of EBER by in situ hybridization (Fig. 2b, c, d). Therefore, the final pathology was follicular dendritic cell tumor of the liver.

## Discussion

Dendritic cells with dendritic morphology belong to immune system, which are classified into two types of cells, interdigitating dendritic cells and follicular dendritic cells. They both are morphologically similar but functionally quite different. Follicular dendritic cells are present in the germinal centers of lymphoid follicles in the spleen and lymph nodes and hence are termed follicular dendritic cells. In the liver, they are described around portal spaces. They are functionally as antigen-presenting cells and thus improving the quality of the humoral immune response [1]. Follicular dendritic cells tumor, which was first described in the literature in 1986 in a series of four cases [2], approximately 100 cases have been described in the literature [3, 4]. In 1996, Shek et al described the first case of FDC tumor of the liver [5]. Since then, less than 20 cases of hepatic follicular dendritic cell tumor have been reported. A total of 16 cases of FDC tumor with hepatic involvement were retrieved from the literature through Medline/PubMed. Clinical information on these 16 cases, along with the current case was detailed in Table 1.

**Table 1:** The Clinical Manifestation of Collective Cases With FDC Tumor

Case	Age (y/o)	Gender	Clinical Presentation	Maximal size (cm)	EBV	Treatment	Reference
1	68	F	Malaise, weight loss, anemia	11	+	Chemotherapy; partial hepatectomy	[11]
2	35	F	Epigastric discomfort, fever, weight loss	20	+	Right hemihepatectomy	[5]
3	37	M	Malaise, weight loss	15	+	Right trisegmentectomy with caudate lobectomy	[16]
4	19	F	R't upper quadrant pain, weight loss, palpable abdominal mass	12	+	Excision	[18]
5	56	F	Gastrointestinal upset	15	+	Resection	[18]
6	40	F	Epigastric pain, weight loss	12.5	+	Left hepatectomy	[18]
7	49	F	Liver mass on ultrasound during routine health check-up	4.2	+	Excision	[18]
8	31	F	Abdominal distention, weight loss	15	+	Right hemihepatectomy	[18]
9	57	F	Epigastralgia	9.5	+	Right hemihepatectomy	[17]
10	51	F	Abdominal discomfort, weight loss	12	+	Left lobectomy	[18]
11	30	F	Liver mass on ultrasound during routine health check-up	5.5	+	Right lobectomy	[19]
12	82	M	Liver mass on abdominal CT scan during evaluation of renal calculi; weight loss, weakness	10	-	Right lobectomy	[21]
13	57	F	Abdominal pain, dizziness, vomiting	NA	+	Resection	[4]
*14	70	M	Abdominal pain, weight loss	2.5	-	NA	[6]
*15	65	F	Staging workup showed lesion in liver from abdominal CT	2	NA	Resection	[7]
*16	19	M	Right upper quadrant pain	16	NA	Chemotherapy^#^	[8]
17	36	F	Epigastric discomfort	14	+	Left Lateral segmental hepatectomy	Present case

+: positive; -: negative; NA: Not available; *:the metastatic cases; EBV: Epstein-Barr Virus; #: well response to chemotherapy.

The mean age of the 17 patients was 47 years old with a wide range from 19 to 82 years old. The disease was much more common in females than in males (13:4). Torres et al had the similar observation report [6]. The clinical manifestations of these patients included abdominal discomfort, palpable mass, weight loss or malaise. The average size of the tumor was about 11 cm. Thirteen of the cases (76.5%, 13/17) were associated with Epstein-Barr virus expression, positive for EBER. Two cases were presented as a metastatic lesion with smaller size of the tumor [7, 8]. Only one of the 17 cases showed no expression of the Epstein-Barr virus [7]. The primary treatment strategy for these patients was surgical resection of the tumor involvement. One case of liver FDC with multiple lymphadenopathies in the celiac and mesenteric retroperitoneal regions showed response to chemotherapy [9].

FDC tumors are very uncommon neoplasms that may arise in lymph nodes and extranodal sites. These tumors have a heterogeneous histology with spindle-shaped tumor cells that possess variable degrees of pleomorphism and arrange in whorly, fascicular, or storiform patterns. There is a characteristic immunohistochemical profile for FDC tumors. Besides CD21, CD23, and CD35, there are other monoclonal antibodies specific for FDC, such as R4/23, Ki-M4, Ki-FDC1p, and CNA.42. The tumor usually stains for vimentin and SMA, and can be weakly positive for S-100 protein [4]. A new immunohistochemical marker, podoplanin (D2-40) has shown with high sensitivity for FDC tumors and may be useful to help confirm the diagnosis in conjunction with conventional FDC markers, particularly in the differential diagnosis of dendritic cell and histiocytic lesions [10, 11]. The differential diagnosis of hepatic FDC tumors is important, especially from inflammatory pseudotumor (IPT) of liver. IPT is a non-neoplastic condition of unknown etiology with myofibroblastic or fibrohistiocytic nature. Some of the IPT have been reclassified as FDC tumors later. However, IPT did not contain cells with marked pleomorphism, and the spindle cells did not express FDC markers. FDC tumors also lack expression of CD1a, CD68, desmin, and CD45, allowing their differential diagnosis with interdigitating dendritic cell tumors, Langherhans cell tumors, histiocytic and lymphoid neoplasias, primary or metastatic sarcomas to the liver, like leiomyosarcomas, fibrosarcomas, or gastrointestinal stromal tumors [4].

The etiology of the hepatic FDC tumor remains unclear. No specific risk factors could be elaborated. The high association between the EBV expression and the hepatic FDC tumor may need further investigation [5, 12-14]. Evidence for the role of EBV in the pathogenesis of the FDC tumor may be needed includes: (a) elevated antibody titers to the virus preceding development of the neoplasm; (b) presence of the viral genome within the neoplastic cells but not in associated/adjacent nonneoplastic cells; (c) clonality of the viral genome; and (d) expression of viral genes in the neoplastic cells [15].

Various treatments including surgery, radiation therapy, chemotherapy and even target therapy have been reported [3, 5, 9, 12, 16-20]. Surgical treatment remains the mainstay of the treatment, however, the optimal treatment or combination treatment for the FDC tumor has yet to be defined due to the limited experience. The prognosis of the hepatic tumor is unclear, mainly due to small number of cases and the retrospective nature of the reports. They are considered low-grade sarcomas from the classification of World Health Organization [21]. In a review of reported cases limited to extranodal FDC tumor found the 2- and 5-year recurrence-free survival rates to be 62.3% and 27.4% respectively [13]. Intra-abdominal occurrence has been associated with a poor prognosis. Literature review showed intra-abdominal involvement, a high mitotic figure (≥ 5/10 high power field), coagulative necrosis and significant cellular atypia are potentially helpful morphological predictors of unfavorable outcome. [13, 22] In conclusion, hepatic follicular dendritic cell tumor is a rare entity. Further investigation about the etiology, treatment modality and prognostic factors of the disease are needed.
